# Spontaneous network transitions predict somatosensory perception

**DOI:** 10.1093/cercor/bhaf309

**Published:** 2025-11-26

**Authors:** Abhinav Sharma, Joachim Lange, Diego Vidaurre, Esther Florin

**Affiliations:** Institute for Clinical Neuroscience and Medical Psychology Medical Faculty, Heinrich-Heine University, Universitätsstr. 1, 40225 Düsseldorf, Germany; MRC Brain Network Dynamics Unit, University of Oxford, Mansfield Rd, Oxford OX1 3TH, United Kingdom; Nuffield Department of Clinical Neurosciences, University of Oxford, Level 6 West Wing, John Radcliffe Hospital, Oxford OX3 9DU, United Kingdom; Institute for Clinical Neuroscience and Medical Psychology Medical Faculty, Heinrich-Heine University, Universitätsstr. 1, 40225 Düsseldorf, Germany; Department of Clinical Medicine, Center of Functionally Integrative Neuroscience, Aarhus University, Universitetsbyen 3, Building 1710, 8000 Aarhus C, Denmark; Department of Psychiatry, University of Oxford, Warneford Hospital, Oxford, OX3 7JX, United Kingdom; Institute for Clinical Neuroscience and Medical Psychology Medical Faculty, Heinrich-Heine University, Universitätsstr. 1, 40225 Düsseldorf, Germany; McGovern Institute for Brain Research, Massachusetts Institute of Technology, MIT Bldg 46-3160, Cambridge, MA 02139, United States

**Keywords:** coherence, connectivity, MEG, spontaneous whole-brain networks, tactile perception

## Abstract

Sensory perception is essential for transforming incoming information in the brain into targeted behavior. Our brains are everlastingly active, and variations in perception are ubiquitously associated with human behavioral performance. Previous studies indicate that changes in spontaneous neural activity within local sensory areas correlate with the perception of ambiguous stimuli. However, the contribution of whole brain spontaneous networks to perception is not well understood. Using an ambiguous tactile temporal discrimination task, we demonstrate that the interaction between whole-brain networks in the seconds of the spontaneous prestimulus period also contributes to perception during the task. Transitions to a frontal and a multifrequency network across the brain are essential for the correct percept. Conversely, incorrect percepts are mainly preceded by transitions to an alpha-parietal network. Brain switches occur faster during the period before stimulus presentation for correct stimuli detection, suggesting the need for enhanced network flexibility during this phase.

## Introduction

Humans are not constantly engaged in tasks. But even at rest, our brains are buzzing with activity, not about any specific cognitive demand, called spontaneous activity. This spontaneous activity is not mere noise, as highlighted by the research on resting state networks (Lewis et al. 2009; Mennes et al. 2010; Northoff et al. 2010; Mastrovito 2013). Rather, spontaneous activity might influence our perception and decisions. Compare your daily morning routine over two days. Even if everything remains the same, you will produce variability (good or bad) across tasks on different days. To understand such variability, it is important to understand the relevance of spontaneous neural fluctuations.

There is already some knowledge about fluctuations within local brain areas during the prestimulus period. Perception correlates with changes in neural activity within brain regions processing sensory information milliseconds before a task begins (Linkenkaer-Hansen et al. 2004; Baumgarten et al. 2016; Rajagovindan and Ding 2011; Iemi et al. 2019; Podvalny et al. 2019; Rassi et al. 2019; Li et al. 2020). Since local brain areas do not work by themselves, this knowledge is likely only the tip of the iceberg. Rather, whole brain networks are likely to be at play and influence the activity in local areas. Time-resolved techniques robustly identifying whole-brain networks from spontaneous data indicate that our brain at rest switches between networks every 50–200 ms (Baker et al. 2014; Vidaurre et al. 2018b). Therefore, differences in spontaneous network configurations and their temporal interplay during an extended period before a stimulus presentation might be responsible for perception differences.

While previous studies have significantly advanced our understanding of regional oscillatory dynamics, such as prestimulus alpha power and phase predicting perceptual variability (Baumgarten et al. 2016; Iemi et al. 2019), they often emphasize localized excitability states. In contrast, we sought to complement this approach by investigating how transitions between distributed whole-brain networks unfold during extended prestimulus periods and contribute to perception. Our focus on spontaneous network dynamics thus extends the scale of analysis, while remaining compatible with the established regional findings.

**Fig. 1:**
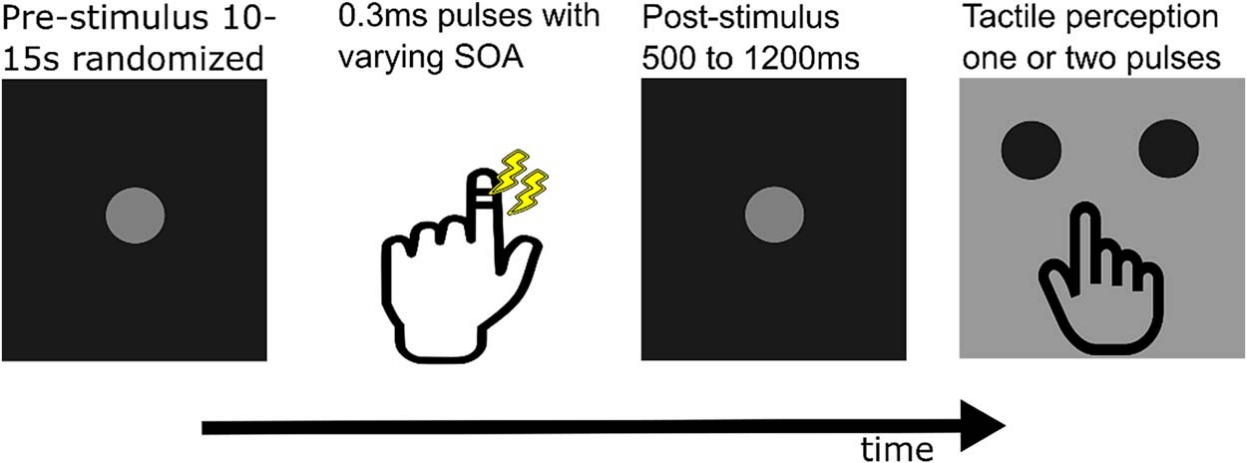
Schematic of the task paradigm (see Methods: Tactile discrimination paradigm), which was adapted based on previous studies (Baumgarten et al. 2015 & 2016). The presented stimulus onset asynchrony (SOA) varied between 0 ms, the critical SOA, critical SOA ± 10 ms, and 3 times the critical SOA.

We investigated the relevance of whole-brain networks during the extended prestimulus phase for tactile perception using an ambiguous tactile temporal discrimination task in the Magneto-encephalogram (MEG). Participants received two short consecutive electric tactile stimuli. They indicated with a button press the percept of either one or two pulses (Fig. 1). Short inter-stimulus intervals are used to induce perceptual ambiguity because they cause the perception of only one stimulus despite two being applied. Using a staircase procedure, we determined the critical stimulus onset asynchrony (SOA) for each participant, ie the inter-stimulus interval inducing 50% correct detections of two stimuli. Using a data-driven approach (Vidaurre et al. 2018a, 2018b) in the form of a Hidden Markov Model (HMM) on the seconds of electrophysiological spontaneous brain activity before trials with ambiguous percept, we identified whole-brain network properties and interactions that predict each individual stimulus percept. We hypothesize that correct and incorrect detection involve different temporal configurations of the pretrial whole-brain networks. Previous studies investigating tactile processing attribute an important role to the fronto-parietal cortices in complex tactile perceptual decision-making (for a review, see Romo and Rossi-Pool (2020)). Hence, we focus on how fronto-parietal connections with brain regions primarily involved in tactile perception, ie sensorimotor cortices, and their temporal evolution cause trial-by-trial variability in tactile perception.

Our findings show two distinct whole-brain transition patterns related to correct and incorrect percepts, demonstrating the role of the interconnection and temporal interplay of spontaneous network configurations across the whole brain during the extended period before a task. This finding indicates that the literature on the neural mechanisms of human perception needs to include the role of dynamic whole-brain network patterns before a task.

## Methods

### Tactile discrimination paradigm

Thirty healthy participants (15 females and 15 males, right-handed, 26.4 (mean), 25 (median), 13 (range) years in age) volunteered to perform the task. The experimental procedure was explained to all participants. Everyone provided written consent, and the study was approved by the local ethics committee at the Medical Faculty of Heinrich Heine University, Düsseldorf (study number 2019–477) and conducted in accordance with the Declaration of Helsinki.

We adopted the tactile discrimination task described in detail in Baumgarten et al. (2016) to accommodate our network analysis. Each trial started with the presentation of a start cue (500 ms). Next, the cue decreased in brightness, indicating the prestimulus period (randomized to last between 10–15 s), after which the subjects received either one or two short (0.3 ms) electrical pulses applied by two electrodes placed between the two distal joints of the left index finger. The relatively long prestimulus period (randomized between 10 and 15 s) was chosen to allow us to model the evolving history of spontaneous neural activity using the HMM. This approach leverages the model’s state-space structure and Markov assumption to track how preceding network transitions unfold over time and ultimately influence perception closer to stimulus onset. Thus, while our core analysis spans 8 s prior to stimulation, this design enables capturing both long-range and immediate influences on perceptual outcome. The time between the two pulses (Stimulus Onset Asynchrony (SOA)) was determined with a staircase procedure for each participant prior to the main experiment so that they had a hit rate of 50%. This SOA is termed the *critical SOA* (crit SOA). Participants had to respond to whether they perceived one or two stimuli with their right hand by pressing a button with either their right index or middle finger. The index and middle finger options were randomly swapped at each trial. SOAs were varied in a pseudorandom manner between: 0 ms, crit SOA, crit SOA + 10 ms, crit SOA—10 ms, and 3 times crit SOA. The 0 and 3 times crit SOA trials were used to prevent biased responses and to be able to confirm that the participants were properly performing the task. Specifically, the 0 ms SOA trials are expected to be perceived as one pulse, and the 3× crit SOA trials as two distinct pulses. Consistent and accurate responses to these unambiguous trials served as behavioral checks to verify participants’ understanding and engagement. Participants were recorded on 2 days: Day 1 for each participant started with a pretask eyes open resting state MEG recording for 10 min. It continued with the staircase procedure and the MEG data recordings during the task. The task was divided into four blocks per day of about 45–50 trials. Each block lasted about 10–15 min. Participants were allowed to take breaks between blocks. Finally, a 10-min post-task eyes-open resting state recording was performed.

On day two, the staircase procedure was repeated. This was followed by four measurement blocks of tasks during which MEG data were recorded. In total, across the two days, 200 trials with crit SOA, 50 of the other four conditions were acquired per participant. On the first day, crit SOA across subjects was 71 ms (mean) + − 34.3 ms (standard deviation); on the second day, crit SOA across subjects was 79.8 ms (mean) + − 34.0 ms (standard deviation). The overall average crit SOA was 75 ms (mean) + − 34.0 ms (standard deviation). The accuracy across different SOAs is shown in Supplementary Fig. S7.

### Electrophysiological recording

MEG data were recorded on both days of task performance. We used a whole-head MEG system with 306 channels (Elekta Vectorview, Elekta Neuromag, Finland) housed within a magnetically shielded chamber. Electrooculography and electrocardiography were recorded simultaneously with MEG data to be later able to remove eye blink and cardiac artifacts. Data were acquired at a sampling rate of 2,500 Hz with an 833 Hz low pass filter. The details of MEG-data preprocessing and source reconstruction can be found in the Supplementary Methods.

### TDE-HMM pipeline

The HMM is a data-driven probabilistic algorithm that finds recurrent network patterns in a multivariate time series (Vidaurre et al. 2018a; Sharma et al. 2021). Each network pattern is referred to as a “state” in the HMM framework, such that these networks can activate or deactivate at various points in time. We use network throughout the text when referring to an HMM state. We used a specific variety of the HMM, the time-delay embedded (TDE)-HMM, where whole-brain networks are defined in terms of spectral power and phase coupling (Vidaurre et al. 2018b). For every point in time, the HMM algorithm provides the probability that a network is active. In our approach, we also performed spectral analyses of these networks, leading to a complete spatio-spectral connectivity profile across the cortex. A summary of the MEG data processing is provided in Supplementary Fig. S8. A TDE-HMM was used to identify recurring whole-brain network patterns across trials. The embedding was performed using a 60 ms window (15 samples at 250 Hz), and the dimensionality of the resulting data was reduced via PCA to 84 components (42 cortical parcels × 2). We fitted a single HMM model across all participants and trials in which the SOA fell within the critical SOA ± 10 ms range, irrespective of perceptual outcome. This window was selected to isolate ambiguous perceptual processing. Pre-stimulus data spanning from −8.0 to −0.1 s relative to stimulus onset were used for training the model. Resting-state or full continuous task data were not included. Based on previous work and pilot analyses, we set the number of HMM states (networks) to six, balancing between model complexity and spectral clarity. Detailed information regarding the HMM fitting can be found in the Supplementary Information.

We preselected four of the six identified networks for further analysis to avoid redundancy and overfitting in our interpretations. The inclusion criteria were based primarily on the coherence ring figures, which allowed us to identify networks with distinct spectral and spatial connectivity features. We retained networks that (i) exhibited spatial specificity with strong coherence in a single frequency band (eg alpha-parietal and high-beta/gamma networks), or (ii) exhibited multifrequency coherence with broad connectivity across cortical areas (eg cross-brain and frontal networks). The two excluded networks demonstrated sparser and more spatially disorganized connections, lacking clear frequency specificity, or dense topological clustering. These networks also did not show any percept-specific temporal dynamics (ie transition rates, lifetime, or fractional occupancy (FO)). Hence, they were considered functionally redundant relative to the selected networks and excluded to preserve our interpretation's parsimony.

### Group-level coherence ring figures

To separate background noise from the most robust coherent connections, a Gaussian mixture model (GMM) was used (Vidaurre et al. 2018b). For the group-level coherence projections, we normalized the activity in each network for each spectral band by subtracting the mean coherence within each frequency mode across all networks. As a prior for the mixture model, we used two single-dimensional Gaussian distributions with unit variance: one mixture component to capture noise and the other to capture the significant connections. This GMM with two mixtures was applied to the coherence values of each network. Connections were considered significant if their p-value after correction for multiple comparisons (Bonferroni correction) was smaller than 0.001.

### Connectivity manifolds via diffusion maps

To better interpret the network structures identified by the HMM, we employed diffusion map embedding to recover low-dimensional representations of network-specific cortical connectivity patterns. This method accounts for both direct and higher-order relationships between brain regions, offering a more integrative view of functional connectivity beyond pairwise coherence measures. Diffusion maps were computed for each HMM network by constructing similarity graphs based on the covariance structure between brain regions, followed by eigen-decomposition of the diffusion operator. The resulting low-dimensional embeddings, termed connectivity manifolds, allowed us to characterize whether networks played sparse or dense roles in brain-wide communication. A detailed description of the diffusion map algorithm and its implementation is provided in the Supplementary Information.

### Statistical testing on the connectivity manifold

The connectivity manifold indicates the distance between different brain regions. In short, the smaller the distance between two regions on the manifold, the stronger the connectivity between the two regions (direct and indirect combined). For details about the relationship between distance and connectivity, please refer to Methods: Connectivity manifolds via diffusion maps. To test if certain brain regions showed significant connectivity on the manifold, we applied the same test described in Methods: Group level coherence ring figures.

### Transition matrix test

We estimated transition matrices for each trial. For every trial, a transition matrix contains probabilities for different HMM networks to switch to and from one another. Non-parametric permutation testing (5,000 permutations) was performed to compare network transitions between correct and incorrect trials. Results were considered significant if *P* < 0.05 after correction for multiple comparisons using the Benjamini Hochberg FDR correction (Benjamini and Hochberg 1995).

### Temporal properties

To test for changes in the temporal properties for percept one versus percept two stimuli, we compared the lifetimes and FO for each network within and across the response types using two-way repeated measures ANOVA followed by post-hoc tests. A network’s lifetime for a given visit refers to the time spent in that network. Of note, the lifetime of a network can be shorter than one oscillation cycle because the frequency is the rate of change of a signal, and this rate can be estimated by examining the slope of the signal at any instant in time (Vidaurre et al. 2018b). FO is defined as the average share of time spent in a particular network. Finally, we assessed the overall network changes per second which is defined as the number of times one network switches to another per second.

## Results

Using a TDE-HMM, we analyzed the spontaneous formation of whole-brain neural networks. Within the 8 s before the tactile stimulation, we identified six distinct networks. These networks were characterized by their spatial distribution and spectral fingerprint. Crucially, we found that the brain spontaneously switches between these networks and that the network active at the moment of stimulation correlates with participants’ perception. In the first step, we will spatially and spectrally characterize the static network properties. These properties are, in turn, relevant for interpreting the dynamic properties of the networks associated with differing percepts.

### Spontaneous prestimulus activity organizes into distinct spectral networks

We identified six whole-brain networks with an HMM from the prestimulus time period of all critical SOA and critical SOA ± 10 ms trials regardless of the percept. We defined 42 cortical regions based on the Mindboggle atlas for further spectral and connectivity characterization. For the spectral characterization, we determined data-driven main frequency modes (see Supplementary Fig. S1 and Supplementary Methods), which entail the alpha, beta, and high-beta/gamma bands. In contrast to canonical bands, these frequency modes allow for subject-specific weights of each frequency bin within a band.

**Fig. 2:**
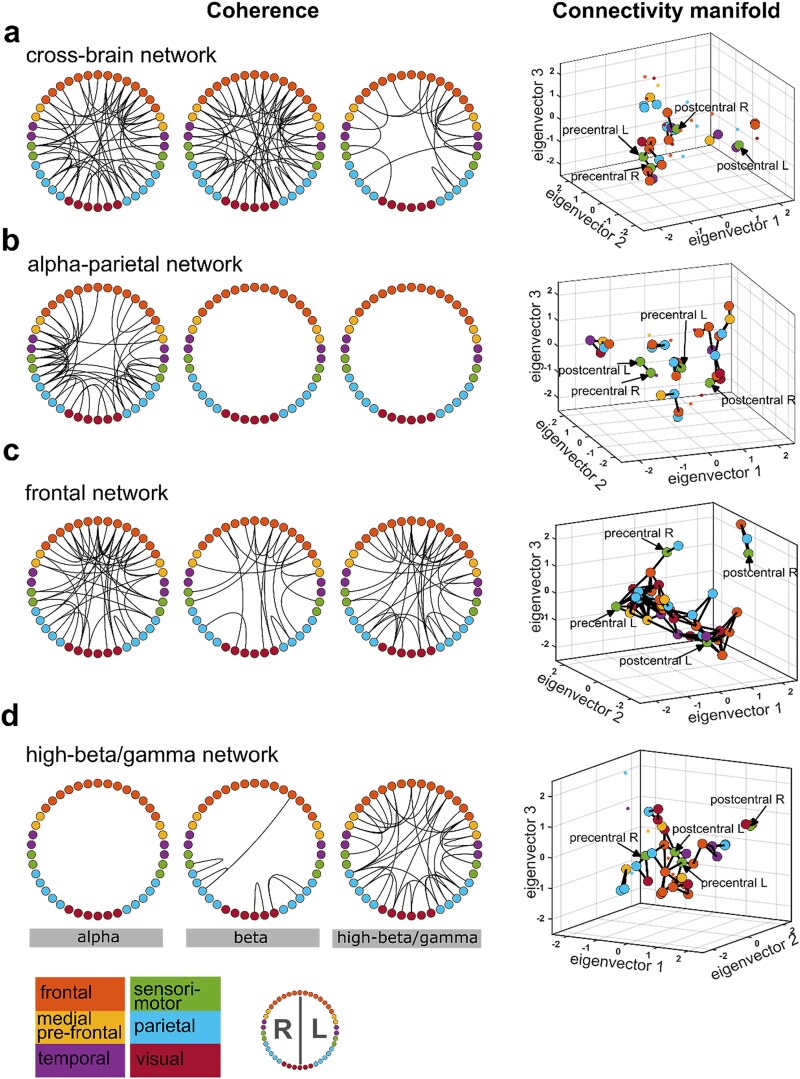
Coherence and connectivity of the four main networks. Each dot in the coherence ring and the connectivity manifold represents one of 42 cortical areas and is color-coded for the main brain region. The exact cortical areas are depicted in Supplementary Fig. S2. a) Cross-brain network. The connectivity manifold for the cross-brain network indicated connections of the left and right precentral (motor cortices) with the frontal and parietal brain regions. b) Alpha-parietal network. This network was characterized by posterior alpha band coherence, but parietal cortex node connectivity was fragmented into different clusters. c) Frontal network. Frontal regions were coherent both within frontal cortex regions and to different brain regions across all three frequency bands. This network is densely connected within one main cluster. d) High-beta/gamma network. Significant connections were only present in the high-beta/gamma band both within the frontal and parietal regions as well as between the regions. The frontal and parietal regions form a cluster with left post-(somatosensory) and pre-(motor) central gyri.

First, we found one network that spans the whole brain, including frontal, temporal, and parietal areas (Fig. 2a, left part). This network was characterized by connections in multiple frequency bands, ie the alpha, beta, and high-beta/gamma bands. Interestingly, while alpha and beta-band connectivity is found across virtually all regions, the connectivity is sparser in the high-beta gamma band. Due to the contribution of all cortical areas, we termed this network *cross-brain* network. The second network was dominated by connections within and from the parietal cortex (Fig. 2b). This connectivity was only present in the alpha band. Due to its spatial-spectral characteristics, we termed it the *alpha-parietal network.* The third network was dominated by spectral coherence in the high-beta/gamma and alpha-band, with a prominent concentration of coherent nodes in the frontal and medial prefrontal regions across all three frequency bands. Additionally, coherence within frontal and medial prefrontal regions was denser than in other network parts. These observations motivated the label “frontal network” (Fig. 2c). The fourth network was characterized by high-beta/gamma band coherence between and within frontal and parietal areas and termed *high-beta/gamma network* (Fig. 2d). Importantly, the involvement of sensorimotor areas in each of these four networks suggests that they are indeed task-relevant. Finally, there were two networks with very sparse connectivity, thus putatively irrelevant for tactile perception (see Supplementary Fig. S3). In the following, we focus on the four main networks.

We selected four of the six HMM networks extracted for detailed interpretation based on their distinct spatio-spectral connectivity features. This decision was guided primarily by the coherence ring representations, which revealed whether a network displayed frequency-specific or anatomically structured coherence patterns. The included networks covered either (a) spectrally specific but spatially localized connectivity (eg the alpha-parietal or high-beta/gamma networks) or (b) multifrequency and spatially distributed coherence (eg the cross-brain and frontal networks). The two excluded networks (see Supplementary Fig. S3) showed sparse, fragmented connectivity, lacked frequency or spatial selectivity, and did not display any differentiating temporal dynamics between percepts (see Supplementary Fig. S5 and S6). We therefore focused our analysis on the four most informative and nonredundant networks to maintain interpretability.

### Connectivity clusters in the HMM networks

A brain region might operate simultaneously in different frequencies to perform multiple functions, enabling flexibility in the region's inputs and outputs (Akam and Kullmann 2010, 2014). Our previous section's result indicated that numerous pairs of brain regions are coherent across several frequency modes within the same network. However, certain cortical regions were coherent in identical spectral bands across different networks. We calculated diffusion maps of each network to untangle whether this is because coherence cannot distinguish if a connection between two brain areas is direct or indirect, ie mediated via multiple brain areas (Lian et al. 2015; Margulies et al. 2016). Diffusion maps extract a low-dimensional representation of data while preserving similarity calculated in higher dimensions. Figure 2 visualizes the maps on a three-dimensional connectivity manifold, which accounts for direct and indirect connections and places regions with greater connectivity closer to each other.

The right column of Fig. 2 shows the connectivity manifold of each network. First, for the cross-brain network, only a few brain regions were statistically significantly connected with their neighbors on the cross-brain network’s connectivity manifold, indicating a sparsely connected network. This contrasts with the network’s multifrequency and spatially non specific coherence characteristics. Furthermore, the manifold shows that the parietal and frontal cortical areas, left motor cortex, and medial orbitofrontal regions were sparsely connected to each other (Fig. 2a).

Second, the alpha-parietal network’s connectivity manifold displayed several disconnected clusters involving at least one parietal region. The parietal regions were, however, not connected to the left postcentral and right precentral gyrus (Fig. 2b).

Third, all brain regions within the frontal network were connected in a single cluster, indicating a densely connected network. This stands in sharp contrast to the three other networks. The only exception is the right postcentral gyrus, forming a separate cluster with the superior-frontal and inferior-parietal right gyri (IP-r) (Fig. 2c). Of note, the left and right pre- and postcentral gyri were not connected in the frontal network.

Finally, the high-beta/gamma network showed a significant connection between the left precentral and postcentral gyri (Fig. 2d and Supplementary Fig. S4b for a 2D view of those connections). Like the cross-brain network, the IP-r was connected to the left pre- and postcentral gyri via two frontal areas. The left pre- and postcentral gyri were further embedded within a cluster of significantly connected frontal regions.

To summarize, although the four HMM-derived brain networks all engage sensorimotor, frontal, and parietal regions, each network exhibits distinct patterns of frequency-specific coherence and higher-order connectivity organization, suggesting specialized modes of spontaneous network communication prior to stimulus perception.

### Correct and incorrect detection correlate with different network transitions

We need to rely on dynamic network analysis to test our hypothesis that correct and incorrect stimuli detections involve different temporal configurations of the whole-brain networks during the prestimulus period. The HMM analysis is ideally suited for this purpose as it naturally provides dynamic network features like transition probabilities and the duration spent in each network. To investigate our hypothesis, we examined on a single trial level whether transition probabilities to and from a given brain network significantly differed between correct and incorrect percept trials during the prestimulus period. We test for significantly different transition probabilities by testing which transition probabilities are significantly increased in each percept relative to the other (implying they are decreased in the other condition).

**Fig. 3:**
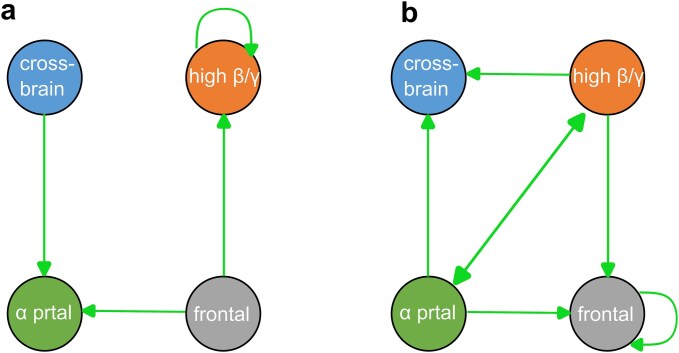
Prestimuli network transition probabilities differing across percepts. The arrows indicate significantly increased network transition probabilities between networks at *P* < 0.05. Not depicted transition probabilities were statistically indistinguishable across percepts. a) Significantly increased network transition probabilities when participants incorrectly detected only one stimulus. b) Significantly increased network transition probabilities when participants correctly detected two stimuli.

For trials in which participants incorrectly perceived one stimulus, the transition probability during the 8-s prestimulus period was higher from the frontal network to the alpha-parietal and the high-beta/gamma network (Fig. 3a, see Supplementary Fig. S5 for the transitions to and from networks five and six). In contrast, the likelihood of correct stimuli detection increased when the transition probability was higher from the alpha-parietal network to the cross-brain, the frontal, and the high-beta gamma network, as well as from the high-beta/gamma network to the cross-brain, the frontal, and the alpha-parietal network (Fig. 3b). The high-beta/gamma and alpha-parietal networks for correctly detected stimuli were the only pair with an increased transition probability in both directions.

### Faster network transitions improve tactile perception

**Fig. 4:**
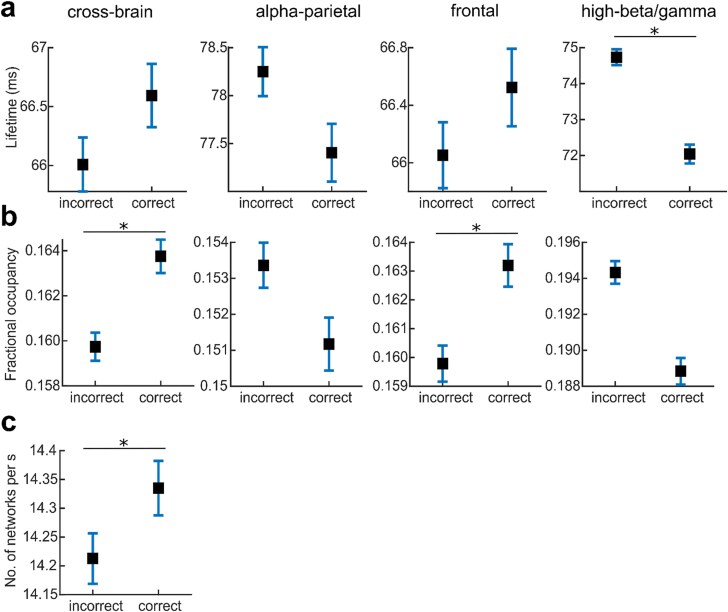
Temporal network properties. Incorrect denotes incorrect stimuli detection trials and correct the correct stimuli detection ones. Box-whiskers represent the mean and standard error across all trials and participants. The stars denote statistically significant results with *P* < 0.01. a) Lifetime for the corresponding HMM network. b) Fractional occupancy (FO) for the corresponding HMM network. c) Number of network visits per second for all six networks. The lifetime and FO of the 2 further networks are presented in Supplementary Fig. S6.

Whether one or two stimuli were perceived crucially depended on the network transitions preceding them. We now focus on three dynamic characteristics of network transitions and investigate their role in accounting for differences in percepts across trials. First, we assess the temporal presence of networks, both in terms of the (i) duration of each visit (lifetime of a network) as well as (ii) the overall share of time spent in a particular network (FO). Second, we consider the rate of inter-network switching, measured by the number of network visits per second during the prestimulus period. Results are shown in Fig. 4.

The lifetime of the alpha-parietal network was the highest (alpha-parietal > all other networks; *P* < 0.001), and its lifetime did not significantly differ between correct and incorrect detections (*P* = 0.59; Fig. 4a). For the high-beta/gamma network, the lifetime and FO were significantly lower for correct than incorrect detections (*P* < 0.001). The cross-brain and the frontal network had a higher FO for correct than incorrect stimulus detections (*P* < 0.001; Fig. 4b). In addition, correct stimulus detections were accompanied by faster overall inter-network switching as the number of network visits per second was significantly increased.

## Discussion

Correctly detecting incoming stimuli in an uncertain, noncued environment requires a particular dynamic pattern of prestimulus spontaneous brain activity that allows the brain to integrate the incoming stimuli optimally. In our work, we first identified four whole-brain networks during the prestimulus resting period that were relevant to the percept. Not all of these networks were simultaneously active, but the switches between those networks correlated with the percept. Correct percepts were characterized by higher transition probabilities from the alpha-parietal and high-beta/gamma networks to the frontal network and lower probabilities to the cross-brain network. In addition, the temporal characteristics of the switches were relevant for the percept: Faster inter-network switching preceded correct stimulus detection, suggesting a need for higher flexibility in the brain.

Our use of a long prestimulus interval deviates from most previous studies that focus on transient dynamics in the subsecond range before stimulus presentation, such as local power or phase modulations. Instead, we sought to investigate whether perception is shaped by a broader temporal context defined by the evolution of spontaneous whole-brain networks. This perspective is enabled by the HMM’s Markovian nature, which allows for tracking sequential network transitions over extended periods. We did not find strong evidence that the activity of any single network at stimulus onset, considered in isolation, reliably predicted perception. Instead, perceptual outcome was linked to how the brain moved between networks across time, particularly transitions into and out of the multifrequency networks. These results indicate that temporal integration across spontaneous networks provides an additional, complementary dimension to the local activation-based mechanisms emphasized in earlier work.

### Multi-frequency networks for correct percepts

Our manifold connectivity analysis revealed two multifrequency networks that support correct perceptual decisions: the cross-brain network and the frontal network. While both depend on multifrequency coherence to coordinate activity across spectral bands, their higher-order topologies diverge sharply. The cross-brain network displays a sparse connectivity manifold, with coherence largely confined to the bilateral precentral gyri and a limited set of fronto-parietal nodes. By contrast, the frontal network forms a dense manifold in which frontal regions maintain widespread multifrequency coupling with the rest of the cortex, which is a pattern well suited for broadcasting multiplexed oscillatory codes (Benchenane et al. 2011; Buschman et al. 2012; Akam and Kullmann 2014; Voytek et al. 2015; Helfrich and Knight 2016; Naud and Sprekeler 2018).

Crucially, the terms “dense” and “sparse” refer only to the relative connection density in each network’s manifold; because spectral coherence and manifold connectivity are undirected measures, we make no claim about the direction of information flow. Together, these findings suggest complementary communication modes: a frontal network that can broadcast multiplexed signals through a dense web of connections, and a cross-brain network that converges information within a more selective, sparsely connected subnetwork. Thus, our terminology captures the distinct topological organization and functional communication patterns inherent in each multifrequency network.

### Spectrally-specific networks in relation to known power changes

In the case of the high-beta/gamma network—a spectrally-specific network—the left post- and precentral regions were embedded within prefrontal areas. Of note is the right IP-r's presence in the prefrontal and left sensory-motor regions’ cluster. The static spectral properties and the dynamic connectivity properties for the high-beta gamma network align with previously reported findings in literature: In the sensorimotor system, beta activity is considered inhibitory (Donner et al. 2009; Pogosyan et al. 2009), while in the prefrontal regions, beta activity tends to indicate reactivation of neuronal ensembles for further processing (Haegens et al. 2011; Spitzer and Haegens 2017; Miller et al. 2018). Moreover, it is known that IP-r in the human brain detects salient stimuli within sequences and controls attention over time (Singh-Curry and Husain 2009; Royal et al. 2016). Hence, the high-beta/gamma network includes an ensemble of fronto-parietal cortical regions that activate perceptually relevant sensory-motor regions.

The spectral characteristics and the connectivity of the alpha-parietal network are consistent with the modulating role of parietal alpha activity for attention (Klimesch 2012; Vollebregt et al. 2016; Helfrich et al. 2017). Dense pair-wise alpha-band coherence between brain regions in the alpha-parietal network exhibited sparse connectivity dominated by parietal areas. The sparse parietal connectivity fits with the described role of posterior alpha activity in gating cortical processing (Jensen and Mazaheri 2010; Foxe and Snyder 2011; Zhou et al. 2021). Furthermore, the lifetime of the alpha-parietal network was the longest among all networks and did not significantly differ between correct and incorrect stimuli detections. Thus, the alpha-parietal network was not suppressed for correct stimuli detections, in contrast to the well-described task-related alpha power suppression in posterior areas (Jensen and Mazaheri 2010; Foxe and Snyder 2011; Klimesch 2012; Arana et al. 2022). Instead, for correct stimuli detection, transitions occur back and forth between the alpha-parietal network and the high-beta/gamma network, and the alpha-parietal network has a higher probability of transitioning to multifrequency networks. These observations highlight a complementary mechanism to traditional models: rather than requiring suppression of posterior alpha per se, perceptual facilitation may involve the flexible reconfiguration of alpha-dominated networks toward multifrequency, task-supportive states. Although we did not directly compare classical alpha-phase effects within our dataset, we did examine prestimulus power changes (see Supplementary Information, Methods: Time–Frequency Analysis) in the right post- and precentral gyri, contralateral to the stimulated finger. Alpha power in the right postcentral gyrus (primary somatosensory cortex) was significantly elevated from −1.0 s to −0.5 s before stimulus onset (Supplementary Fig. S10; *P* = 0.014). Higher prestimulus alpha activity in this region increased the likelihood that participants would misperceive two pulses as a single pulse. This pattern mirrors the findings of Baumgarten et al. (2016), who employed the same task with a shorter inter-stimulus interval. Thus, our findings suggest that large-scale network dynamics offer an additional explanatory dimension alongside local oscillatory modulations. Future studies could explicitly contrast these levels of description to elucidate their respective contributions to perceptual variability.

The absence of a distinct theta-band network in our results warrants further comment. While theta-band activity has been widely linked to frontal cognitive control and attention (Cavanagh and Frank 2014; Rajan et al. 2019; Adamczyk and Wyczesany 2023), our spectral decomposition did not yield a separate theta mode. We used a data-driven NNMF approach to extract dominant spectral profiles from the coherence matrices, which identified three oscillatory modes corresponding to alpha, beta, and high-beta/gamma, and a fourth low-frequency mode consistent with 1/f aperiodic activity. This broadband component encompassed the theta range but lacked spectral specificity and was therefore excluded from oscillatory interpretations. It is also possible that the NNMF decomposition, while effective for isolating structured oscillatory modes, was not sufficiently sensitive to finely resolve lower frequencies such that a distinct, stable theta mode could emerge. We view this both as a constraint of the decomposition method and as a reflection of the absence of dominant theta-band coherence across trials.

### Connectivity manifolds for network descriptions

HMM is an unsupervised method that is blind to the underlying biology of the time series on which it is fit. The algorithm is not explicitly built to extract physiologically interpretable features. Hence, the connection between biology and the HMM networks is necessarily post-hoc. In our case, the cross-brain and the frontal network exhibited multifrequency and spatially distributed spectral coherence. This overlap in coherence between the identified networks complicates the interpretation of each network’s physiological relevance. Hence, we used diffusion maps on network-specific covariance vectors of each brain area to capture higher-order connectivity between different brain regions within each HMM network. The low-dimensional data representation of the diffusion maps allowed identifying a brain network’s most critical connections. It enabled us to disentangle the two multifrequency networks by revealing that the cross-brain network operates with a sparser connectivity profile, whereas the frontal network exhibits dense, widespread coupling. Hence, diffusion maps offer a complementary framework to coherence-based whole-brain networks.

### State time courses as predictors of percept

To further probe whether fine-grained, short-timescale state dynamics immediately before stimulus onset could predict perceptual outcome, we conducted an additional time-resolved analysis using the state probability time courses (ie "gammas"). Specifically, we extracted the HMM state time courses across the 600 ms prestimulus period at ~ 60 ms resolution, and used these in a LASSO-regularized logistic regression framework to predict participants’ perceptual decisions. No significant predictive clusters of time points were found across any of the networks (Supplementary Fig. S9). This negative result suggests that perception in our ambiguous tactile task is not predominantly shaped by brief, transient state activations immediately preceding the stimulus. Rather, it supports the idea that longer-term spontaneous network reconfigurations—such as changes in transition rates and broader network switching patterns over several seconds—are key determinants of perceptual variability.

## Conclusion

Over the years, specific brain regions and their spectral properties have been linked to perception. However, brain areas do not operate in isolation but are intensely connected with each other. We highlighted that the likelihood of correctly detecting the tactile stimuli increased when transitions from spectrally-specific to multifrequency networks were more likely during the prestimulus period. These changes in temporal network characteristics indicate that correct stimulus detection requires whole-brain networks to be able to switch more flexibly and reconfigure network connectivity between sensorimotor, frontal, and parietal regions. Our findings collectively underscore the relevance of spontaneously forming whole-brain networks before the task for understanding mechanisms of perception. This extends previous research on near-threshold stimulus detection beyond the relevance of the short preparatory baseline period (Busch et al. 2009; Baumgarten et al. 2015, 2016; Florin et al. 2017; Podvalny et al. 2019) and thus points to the functional relevance of spontaneously forming whole-brain networks.

Our findings suggest that perceptual variability may arise from an interplay between localized spectral properties and the dynamic coordination of distributed networks. Accordingly, integrating both regional oscillatory measures and whole-brain network dynamics will be crucial for a comprehensive understanding of the neural basis of perception.

## Supplementary Material

Supplementary_Material_bhaf310

## Data Availability

Data will be available upon request, and the software is available under https://github.com/FlorinNeuro.
