# Supplementary material for: Spontaneous network transitions predict somatosensory perception
Source: Cereb Cortex. 2025 Nov 26;35(11):bhaf309. doi: 10.1093/cercor/bhaf309 (PMC12649745; doi:10.1093/cercor/bhaf309)
Supplement: Supplementary_Material_bhaf310 [file supplementary_material_bhaf310.docx]

**Excitatory Magnetic Stimulation Increased Interhemispheric Inhibition: A Pharmacological and TMS-EEG Study**

**Running Title: rTMS Enhances Interhemispheric Inhibition**

Yazhen Han^a^, Zhimin Huang^a^, Yujun Wang^a^, Ying Liu^b^, Jiqing He^c^, Zhongming Gao^d^, Paul B Fitzgerald^e^, Zafiris J Daskalakis^f^, Xianwei Che^a,^*

^a^*Centre for Cognition and Brain Disorders, The Affiliated Hospital of Hangzhou Normal University, 2318 Yuhangtang Road, Hangzhou, 311121, China*

^b^*Department of Anesthesiology, The Second Affiliated Hospital of Zhejiang University School of Medicine, 88 Jiefang Road, Shangcheng District, Hangzhou, 310009, China*

^c^*Department of Obstetrics, The Affiliated Hospital of Hangzhou Normal University, 126 Wenzhou Road, Gongshu District, Hangzhou 310015, China*

^d^*Department of Neurology, The Affiliated Hospital of Hangzhou Normal University, 126 Wenzhou Road, Gongshu District, Hangzhou 310011, China*

^e^*School of Medicine and Psychology, Australian National University, Canberra, Australian Capital Territory, ACT 2601, Australia*

^f^*Department of Psychiatry,* *University of California San Diego, 9500 Gilman Drive, La Jolla, CA 92093, USA*

**Corresponding Author**:

Professor Xianwei Che

Fax: 86-571-28867717; Phone: 86-571-28867717; Email: [xwcheswu@gmail.com](mailto:xwcheswu@gmail.com)

Mailing address: Building 19, 2318 Yuhangtang Road, Hangzhou, 311121, Chin

**
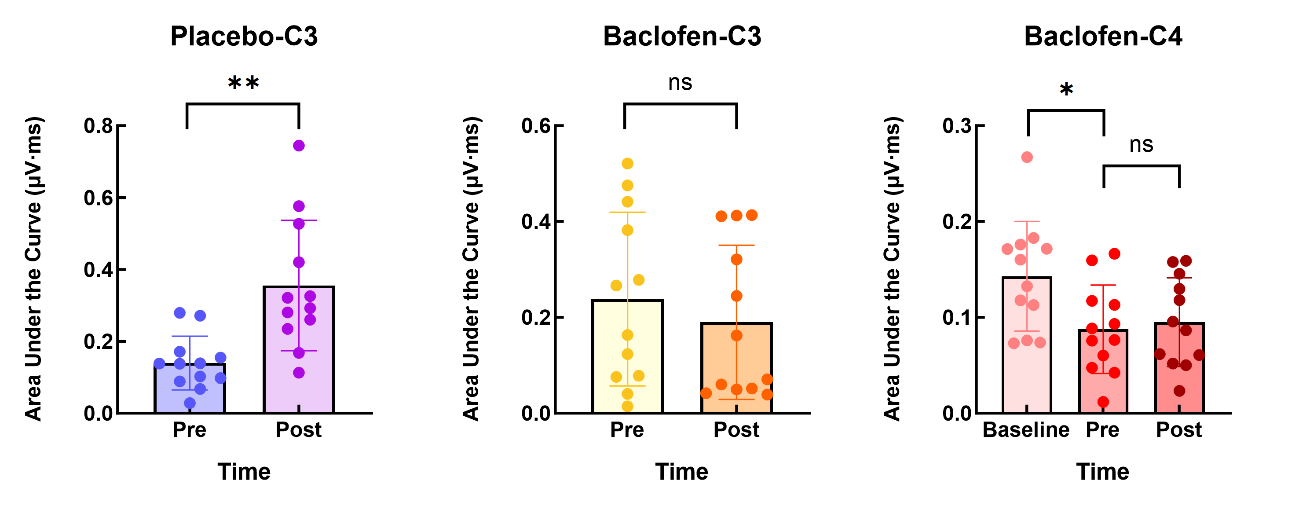
**

**Supplementary Fig. 1.** AUC Changes in Different Conditions. (A) Under the placebo condition, rTMS induced a significant increase in the area under the curve (AUC) in the C3 channel. (B) After baclofen administration, rTMS no longer produced a significant change in AUC in the target region (C3). (C) Baclofen administration significantly reduced AUC in the C4 channel, and subsequent rTMS did not induce further significant changes.
